# Hyperuricaemia, Xanthine Oxidoreductase and Ribosome-Inactivating Proteins from Plants: The Contributions of Fiorenzo Stirpe to Frontline Research

**DOI:** 10.3390/molecules22020206

**Published:** 2017-01-27

**Authors:** Andrea Bolognesi, Massimo Bortolotti, Maria Giulia Battelli, Letizia Polito

**Affiliations:** Department of Experimental, Diagnostic and Specialty Medicine-DIMES, Alma Mater Studiorum, University of Bologna, Via San Giacomo 14, 40126 Bologna, Italy; andrea.bolognesi@unibo.it (A.B.); massimo.bortolotti2@unibo.it (M.B.); letizia.polito@unibo.it (L.P.)

**Keywords:** gout, hyperuricaemia, immunotoxins, ribosome-inactivating proteins, ricin, toxic lectins, xanthine oxidoreductase

## Abstract

The enzymes called ribosome-inactivating proteins (RIPs) that are able to depurinate nucleic acids and arrest vital cellular functions, including protein synthesis, are still a frontline research field, mostly because of their promising medical applications. The contributions of Stirpe to the development of these studies has been one of the most relevant. After a short biographical introduction, an overview is offered of the main results obtained by his investigations during last 55 years on his main research lines: hyperuricaemia, xanthine oxidoreductase and RIPs.

## 1. Biographical Notes

Most of the biographical information that follows was obtained through long-term direct collaboration of some of the authors with Fiorenzo Stirpe. Stirpe was born in Castro dei Volsci (FR) on 28 January 1932, into a cultured middle class family, originally from Ciociaria (near Rome), which, from childhood, stimulated his curiosity and his desire of knowledge. Indeed, he began his studies privately already at the age of four years, showing right away a great desire to learn and the aptitude for scientific subjects. The father, who was a physician, probably transmitted to him the interest for the medical-biological world, an interest that further grew during high school. Afterwards, he enrolled in the school of Medicine at the University of Rome “La Sapienza”, following the family tradition, with the intention to dedicate himself, in the future, to surgery. Anyway, soon after the first contact with biology and its challenges, he matured the idea that this type of study, even if it would not be of service in the practice of medicine, certainly would have been a great basis to undertake what he was beginning to consider as the best job in the world: scientific research. After an internship period at the Institute of Biochemistry, under the guidance of Doriano Cavallini, in 1955, just 23 years old, he obtained the degree of Doctor of Medicine, defending a thesis on the oxidation induced by ascorbic acid. This thesis was awarded Best Thesis of the Year in biological chemistry [1].

After graduation, he became a volunteer assistant at the Institute of Organic Chemistry of the University of Rome. Then he joined the group of Eugenio Bonetti in Messina, at the Institute of General Pathology, where he held the position of Lecturer in General Pathology, from 1957 to 1960. During the time he spent in Messina, he could meditate about the work prospects and he confirmed his choice to devote himself to research. His first experiences abroad date back to those years, when the spent time at Oxford University, in the department directed by Hans A. Krebs, at the Toxicology Unit of the Medical Research Council in Carshalton, and later in the United States at the National Institutes of Health, in Bethesda (MD). These periods spent abroad were very important to the life and career of Stirpe, who, despite his young age, had already developed a strong scientific personality.

In Italy, Stirpe continued his academic career by following Eugenio Bonetti in 1960 to the University of Siena, where in 1961 obtained the position of Senior Lecturer, then, in 1964, to the Institute of General Pathology at the University of Bologna. In this Institute, which later became the Department of Experimental Pathology, Stirpe would spend the rest of his academic life, becoming Associate Professor in 1969 and Full Professor in 1970. He held the position of director of the department from 1986 to 1992.

In the sixties, in Bologna, Luigi Fiume involved him in the study of α-amanitin, the most potent toxin of *Amanita phalloides*. Along with his colleague, Stirpe discovered that the mechanism of action of this molecule consisted in the inhibition of an RNA polymerase, which later would be identified as RNA polymerase II, the enzyme responsible for mRNA synthesis.

During the same period, he continued the study of the enzyme xanthine oxidase, finding that this enzyme is actually a NAD^+^-dependent dehydrogenase, which can be converted into an oxidase. This result allowed other scientists to discover that the oxidase can be responsible for damages to the organism under particular conditions, and opened a research field that has continued until today.

At the beginning of the seventies, Stirpe undertook another research line, which to date continues to stimulate the scientific community and provide a large amount of results. This new research practically coincided with the formation of his own research group. 

This research line led to the identification, purification and characterization of new plant toxins. Some of them, like ricin and abrin, showed a double-chain structure, whilst others were characterized by a single-chain structure. Due to their biological properties, Stirpe named all these toxins “ribosome-inactivating proteins” or RIPs [2]. The research of new RIPs provided new pharmacological molecules for therapeutic purpose. Conjugates of RIP and antibody, for their innovation in experimental therapy, earned Stirpe two prestigious prizes: the “Antonio Feltrinelli” Prize for Medicine, given to him by the Accademia Nazionale dei Lincei, in 1984, and the Pierce Award (USA), in 1995, at the 4th International Symposium on Immunotoxins, in Myrtle Beach, SC.

In 1998, for his prestigious career, Professor Stirpe received the Laurea Honoris Causa in Biological Sciences from the Second University of Naples, and in 2006 he was awarded the title of Professor Emeritus at the University of Bologna—Alma Mater Studiorum.

The scientific production of Fiorenzo Stirpe is documented by his prolific publishing activity. To date, Stirpe has authored 292 publications *in extenso*, including 17 book chapters, nine scientific society advances, and 266 articles published in leading international scientific journals.

## 2. The Fructose Load Test and the Dietetic Control of the Uricaemia

The only article authored by Stirpe about gout was published in 1970 [3]. The results of this research were amazing since they showed a close relationship between the oral assumption of fructose and the serum level of uric acid (SUA) in gouty subjects and in their offspring with an uncontrolled diet. The load test with fructose resulted in a moderate and transient increase of SUA in normal subjects and a marked and persistent increase of SUA in gouty subjects. The hypothesis was formulated that the sensitive reduction of fructose-containing food and drink intake would favourably affect the course of the disease in these subjects. Noteworthy, the sons of these patients showed a time course with intermediate slope between that of healthy and sick subjects in the same test, even in the absence of any symptom.

Thus, further experimentation started with gouty subjects, which showed a pathological response to oral load test with fructose and decided to modify their diet by strongly reducing the consumption of sweet food, fresh fruits and alcoholic beverages. The diet not only caused the normalization of SUA, even in the absence of treatment with allopurinol, and in the presence of a meat-rich diet, but mostly induced the disappearance of pain, without the use of colchicine, and improved the anatomical and functional efficiency of the affected joints.

These results were considered insufficiently proved by The Lancet, which refused their publication, discouraging continuation of this research line. But the huge gratitude to Stirpe aroused in the patients induced him to continue in private. Thus, the experimentation persevered with an increasing number of cases. These data were never published by Stirpe but led only to a publication in a minor Italian magazine, solely authored by De Stefano, one of the coauthors of the previous Stirpe publication on this subject [4].

Hyperuricaemia and gout have a multifactorial aetiology that includes both genetic and environmental causes, thus the fructose load test is necessary to identify the subjects that could form the basis of the therapy on the proposed dietetic limitations. Although the hyperuricaemic effect of fructose has been well established, to date, the fructose load test has not yet been introduced in medical practice and the limitation on fructose intake is only proposed together with a number of other dietary suggestions for the treatment of gout (reviewed in [5,6,7]).

## 3. The Contribution to Studies on Xanthine Oxidoreductase

Only 24 over 292 publications of Stirpe deal with xanthine oxidoreductase (XOR), however they gave an incredible input to the studies of this field, some of them representing major milestones.

The contribution of Stirpe to knowledge on the pathophysiology of XOR started with a study about the diet’s influence on chick-liver xanthine dehydrogenase (XDH) activity [8,9]. However, the breakthrough occurred in 1968 when he observed together with Enrico Della Corte that the activity of xanthine oxidase (XO) dramatically increased in the rat liver supernatant after: (i) storage at −20 °C [10]; or (ii) incubation with proteolytic enzymes [11]. The increase of rat-liver XO activity was also obtained by treatment with solvents or under anaerobic conditions and interpreted as a conversion of the enzyme activity from a dehydrogenase to an oxidase form [12]. Moreover, this XDH to XO change could be reversed with a thiol compound, when obtained by incubating at 37 °C the rat liver supernatant with the sediment, but not when obtained with a proteolytic treatment. The reversible XOR conversion was suggested to occur during its isolation, as a consequence of the enzymatic activity of the microsomal protein-disulphide isomerase [13]. The reversible and irreversible transitions of XOR among its various possible forms was then detailed, confirming that in mammals liver XOR is, in its constitutive form, a dehydrogenase like in birds, but only the mammalian enzyme may undergo such conversion from XDH to XO, possibly including an intermediate XDH/XO form [14]. This conclusion was extended to all rat organs, including intestine, from which XOR could be purified in its dehydrogenase form if the conversion to oxidase was avoided during the enzyme isolation by the addition of thiol compounds and trypsin inhibitor, in the case of intestine, in the purification buffer [15]. Afterward, even cow’s milk XO was purified in a form reversible to XDH by thiols [16].

These findings represented the beginning of a huge amount of research on XOR, performed all over the world, mostly concentrated in the subsequent 40 years and still going on in renewed directions. The interest of Stirpe in studies on XOR, in collaboration with Maria Giulia Battelli, was focused on two peculiar topics: the construction of XOR-antibodies conjugates and the determination of XOR levels in human tissues.

XOR-containing conjugates have been prepared against B [17,18,19,20] and T [21] lymphocytes for the ex vivo purging of bone marrow from haematological malignancies or immunocompetent T cells for autologous or heterologous transplantation, respectively.

The level and type of XOR activity have been determined in human liver both in normal and pathological conditions [22,23]. Also, a competitive enzyme-linked immunosorbent assay (ELISA) was developed to measure the XO level in human serum [24] and used to assess it in human liver disease [25].

## 4. The Mechanism of Action of Ricin

The symptoms of intoxication from castor seeds and pharmacological properties of castor oil have been known for a long time in traditional Indian, Egyptian and Chinese medicine (reviewed in [26]). The modern history of ricin began in the late nineteenth century, dating back to the first description of a toxic haemagglutinin obtained from the seeds of castor bean by Stillmark [27]. In the following years, Paul Erlich, intoxicating animals with ricin or abrin, was able to discover the antibody response. However, even though almost a century had elapsed, in the early 1970s the mechanism of action of ricin and abrin was still unknown.

In those years, Lin and co-workers described a greater toxicity of ricin and abrin to tumour cells compared to normal cells, and the inhibition of protein synthesis in the intoxicated cells [28]. The inhibition of protein synthesis was much more evident in a cell-free system, as later showed by Olsnes and Pihl [29]. These results led Stirpe to study the topic in depth. He thus began a research line that would last throughout his career and that would profoundly affect the scientific community (reviewed in [30]).

Collaborating with Lucio Montanaro and Simonetta Sperti, Stirpe showed that ricin inhibits the elongation of the polypeptide chain dependent on EF2. Together with these scientists, he also showed that ricin does not interfere with the binding of aminoacyl-tRNA to the ribosome and does not damage EF2, but instead acts on the ribosome making it unable to bind EF2. Furthermore, he demonstrated that the toxin was able to damage ribosomes in a less-than-equimolar ratio, an efficiency that could be only explained by the hypothesis of a catalytic activity. Other studies showed that ricin acts by damaging the major ribosomal subunit [31,32]. These results added significant knowledge to the mechanism of action of ricin, allowing Endo, fifteen years later, to clarify the molecular mechanism of the damage to the 28S ribosomal RNA [33].

In collaboration with Luigi Barbieri, Stirpe in 1994 observed that various RIPs from *Saponaria officinalis* are able to depurinate a number of polynucleotide substrates [34], thus identifying for RIPs a wider enzymatic activity for which the name polynucleotide:adenosine glycosylase was proposed [35].

## 5. The Discovery of Type 1 RIPs

In 1973, Olsnes and Phil described the double chain structure of ricin and abrin and the galactose-binding properties of the B chain [36,37]. Trying to find toxins similar to ricin, Stirpe focused its research on poisonous plants, in particular to those belonging to the Euphorbiaceae family, to which *Ricinus communis* also belongs. From the seeds of *Jatropha curcas* and *Croton tiglium*, Stirpe purified protein fractions that were able to inhibit protein synthesis in a cell-free system, but were about 1000-fold less toxic than ricin for animals and cultured cells. These proteins, named curcin and crotin, did not show lectin-like properties and their translational inhibiting activity was not affected by β-mercaptoethanol, thus suggesting a single chain protein structure, as was later confirmed by SDS-PAGE [38].

In a further screening of 27 different plant extracts, 13 were found to inhibit protein synthesis in a cell-free system, but again all resulted much less toxic than ricin towards cells in culture [39]. These results, seemingly unusual, were not immediately understood nor the research was deepened.

A few years later, from *Momordica charantia* seeds, a non-lectin protein with low toxicity to animals and cells was isolated. This protein was much more powerful than ricin and abrin in the inhibition of cell-free protein synthesis [40] and showed a striking resemblance to the pokeweed antiviral protein, a previously described tobacco mosaic virus inhibitor [41]. Both proteins strongly inhibited protein synthesis by inactivating the ribosomes, showed low toxicity to cells and animals, and had a molecular weight similar to that of the A-chain of ricin and abrin. In addition, β-mercaptoethanol did not increase their protein synthesis inhibiting activity. By that time, the idea that single chain proteins similar to the A-chain of ricin, with a strong protein synthesis inhibiting activity, were widespread in the plant kingdom was growing in Stirpe and his group in Bologna. Thus, for the second time, Stirpe and co-workers made a screening of different plant tissues, especially seeds. The results showed that 12 out of 33 different extracts contained proteins able to inhibit protein synthesis [42]. From one of these extracts, during a period of study at the Department of Biochemistry of the Radium General Hospital in Oslo, Stirpe purified gelonin, a protein from the seeds of *Gelonium multiflorum*, which again showed the characteristics of a single-chain inhibitor, able to inactivate the 60S ribosome subunit. The confirmation that the low cytotoxicity of this protein, and perhaps of similar inhibitors, derived from the lack of the lectin chain, was demonstrated by a brilliant experiment, in which Stirpe and the Norwegian colleagues increased by about 3 log the cytotoxicity of gelonin by conjugation to concanavalin A [43].

Other important results were obtained by Stirpe at the Toxicology Unit of the Medical Research Council in London. Here, Stirpe purified two single-chain protein synthesis inhibitors from the leaves of *Dianthus caryophyllus*, namely dianthin 30 and 32 [44]. This plant had attracted attention because its leaf extract had previously been shown to possess antiviral activity against TMV. Indeed, Stirpe verified that many of these plant toxins able to inhibit protein synthesis, such as dianthins, gelonin, momordin, ricin, abrin and modeccin, possess antiviral activity against TMV [45] and some of them (gelonin, dianthin 32 and PAP) also against herpes simplex virus-1 and polio-virus I [46]. This anti-viral activity was then attributed both to their ability of inhibiting protein synthesis and to a direct depurinating effect on viral nucleic acids (reviewed in [47]).

In 1982 Stirpe introduced for the first time the denomination “ribosome-inactivating protein” (RIP) to designate all the protein synthesis inhibitors able to inactivate the 60S ribosome subunit [2]. Stirpe and Barbieri then proposed the designation as type 1 of the single chain RIPs and as type 2 of the RIPs consisting of an A (active) chain, with enzymatic properties, covalently linked to a B (binding) chain, with lectin properties. This was an important milestone in plant toxin research, not only for the first use of the name itself, but also because the existence of a whole class of proteins probably widely distributed in Nature, although at the time still largely unknown, was finally recognized. Equally significant was the placement of the actual toxins, i.e., ricin and others like it, in this category, envisaging their phylogenetic closeness to the single chain inhibitors. The denomination would have been temporary, pending elucidation of the nature of the enzymatic activity of the proteins and their biological function, but the name nevertheless quickly spread and, demonstrating how powerful this insight was, is still used to identify these proteins.

In the following years many other type 1 RIPs were purified at the University of Bologna or in other institutions under the direction of, or in collaboration with Stirpe (see Table 1).

## 6. The Discovery of Type 2 RIPs

After his studies on curcin and crotin, Stirpe turned his attention to *Adenia digitata*, a spontaneous Passifloracea of southern Africa, previously known as *Modecca digitata*. Stirpe had read about this plant in a treaty of medicinal and poisonous plants from Africa. He also read that in 1923 two South African researchers [48,49] had partially identified the toxic components of the plant: a cyanogenic glycoside and a powerful “toxialbumin” that they called modeccin. This toxic protein induced in intoxicated animals pathological changes similar to those caused by ricin. In 1977 Stirpe purified modeccin from the roots of *Modecca digitata* [50] and identified it as a type 2 RIP, because it is able to inhibit protein synthesis by arresting the elongation of peptide chains [50,51,52]. Besides, he described the haemagglutinating activity of modeccin [53], as well as its heterodimeric structure consisting of two subunits with inhibitory and lectin activities [54]. In addition, the toxicity of modeccin to rat was investigated to ascertain the effects on liver ribosomes in vivo [55]. In the same period, other toxicity studies were conducted by Stirpe with ricin in rat to assess the pathogenesis of liver lesion [56], as well as the effect of administration on brain ventricles [57]. Moreover, the selective toxicity of abrin to rat pancreas was detected [58].

The search of Stirpe for other proteins with a ricin-like activity brought him to discover more type 2 RIPs. In the following years, a series of other type 2 RIPs were identified by Stirpe’s group: *Momordica charantia* lectin [40], viscumin from *Viscum album* [59], *Hura crepitans* lectin [60], volkensin from *Adenia volkensii* [61], IRA from *Iris hollandica* [62], Shiga-like toxin I [63], lanceolin and stenodactylin from *Adenia lanceolata* and *Adenia stenodactyla*, respectively [64,65]. Some of these lectins, namely those from *Adenia* plants, are extremely toxic and, in addition, have in common the property of being axonally transported also in central nervous system [66,67,68,69]. This characteristic distinguishes them from other toxic lectins like abrin and ricin that undergo only to retrograde transfer in peripheral nerves [70]. The suicide transport suggests a possible utilization of type 2 RIPs to induce focal neuronal lesion and as agents for immunolesioning in the nervous system [71].

## 7. From Lab Bench to Bedside

In the seventies of the last century, the “magic bullets” of Ehrlich started to become a reality. Hybrid conjugates were synthesized that contain a “haptomer”, able to bind to specific cell membrane receptors and an “effectomer”, able to kill the cell (reviewed in [92]). Stirpe envisaged that type 1 RIPs could be perfect toxic moieties to be selectively delivered to eliminate unwanted cell populations and, in 1980, he conjugated gelonin to concanavalin A. While gelonin alone was not toxic to human lymphocytes, the resulting complex was able to intoxicate them, because the conjugation gave to gelonin the ability of enter the cell [43]. As a carrier to facilitate the entry of type 1 RIPs into cell, Stirpe also used Sendai virus envelopes associated to gelonin, *Momordica charantia* inhibitor or pokeweed antiviral protein, and he obtained a new type of cytotoxic agent against erythroleukaemic cells [93].

The development of monoclonal antibody technology allowed a strong acceleration in anticancer studies with antibody-linked cytotoxic agents. Such chimeric proteins were named immunotoxins (ITs, reviewed in [94]). In 1981, Stirpe and Thorpe conjugated gelonin to a monoclonal antibody against a T cells antigen [95]. Thus, the first IT with a type 1 RIP was achieved, contributing to the implementation of immunotherapy with anticancer purposes. A few years later, ITs made of the same antibody conjugated with saporin [96], bryodin or momordin [97] were prepared. The saporin-containing IT showed a higher power in comparison to the ricin-containing IT against T-lymphoma cells and significantly delayed the neoplastic growth in nude mice, hence prolonging the life of tumour-bearing animals.

Moreover, Stirpe experimented with in vivo bispecific F(ab’γ)_2_ antibody that recognized both saporin and guinea pig lymphoblastic cells. The treatment efficiently delivered saporin to lymphoma cells and completely eradicate tumours in leukemic guinea pigs [98].

In parallel, Stirpe conducted a series of toxicological studies to investigate the aspecific toxicity of ITs for various organs, in particularly for the liver [99,100,101,102,103]. Moreover, the cytotoxic mechanisms of ITs have been explored, showing that cells are killed by the induction of apoptosis [104] as a consequence of the depurinating activity of the RIP moiety [105].

Including the publications about XOR-containing ITs, Stirpe authored 66 articles describing conjugates and their possible medical applications. Most of the type 1 RIPs-containing ITs were produced, in collaboration with Andrea Bolognesi, against B or T-lymphocytes with the purpose of preventing the graft versus host disease in allogenic bone marrow transplantation or treating haematological malignancies. ITs were designed also for the experimental therapy of solid tumours and for the nano-surgery of oculo-motility disorders. (Table 2).

The most encouraging results were obtained in advanced multiple myeloma patients receiving autologous transplantation of IT-purged bone marrow [106,107] and in patients with advanced refractory Hodgkin’s disease treated with an IT prepared by covalently linking saporin to an anti-CD30 monoclonal antibody [108].

## 8. Conclusions

Stirpe contributed to a high degree in a wide range of topics about the RIPs from plants. As underlined in Table 1, an astonishing number of RIPs has been detected, purified and characterized by his research group. He also made impressive contributions to the development of ITs, in particular to their role as anticancer drugs, shown in Table 2. In addition, with restless activity, he investigated the catalytic mechanism of RIPs, their antiviral and abortifacient activities, as well as the possible exploitation in neurobiology and their biological role. The most surprising thing in Stirpe is the ability to nurture heterogeneous interests, and especially the fact that in each of the areas that he has studied he was able to capture profoundly innovative aspects, like the role of fructose in hyperuricaemia, the dehydrogenase activity of mammalian XOR and the therapeutic perspectives of RIP applications.

## Figures and Tables

**Table 1:** RIPs isolated and characterized by Stirpe and co-workers.

Familia/Species	Tissues	RIP	Type	Ref.
**Asparagaceae**				
*Asparagus officinalis*	seeds	asparin 1, 2	Type 1	[72]
**Basellaceae**				
*Basella rubra*	seeds	*Basella rubra* RIP 2, RIP 3	Type 1	[73]
**Caryophyllaceae**				
*Agrostemma githago*	seeds	agrostin 2, 5, 6	Type 1	[72]
*Dianthus caryophyllus*	leaves	dianthin 30, 32	Type 1	[74]
*Lychnis chalcedonica*	seeds	lychnin	Type 1	[75]
*Saponaria ocymoides*	seeds	ocymoidin	Type 1	[76]
*Saponaria officinalis*	seeds	saporin-S5, -S6, -S8, -S9	Type 1	[72,77]
*Saponaria officinalis*	leaves	saporin-L1, -L2	Type 1	[77]
*Saponaria officinalis*	roots	saporin R1, R2, R3	Type 1	[77]
*Vaccaria pyramidata*	seeds	pyramidatin	Type 1	[76]
**Cucurbitaceae**				
*Bryonia dioica*	leaves	bryodin-L	Type 1	[75]
*Bryonia dioica*	roots	bryodin 1, 2	Type 1	[78]
*Citrullus colocynthis*	seeds	colocin 1, 2	Type 1	[75]
*Cucurbita moschata*	seeds	*Cucurbita moschata* RIP	Type 1	[79]
*Momordica charantia*	seeds	momordin I, momorcharin	Type 1	[74,80]
*Momordica charantia*	seeds	MCL	Type 2	[40]
*Momordica cochinchinensis*	seeds	momorcochin-S	Type 1	[81]
*Trichosanthes kirilowii*	seeds	trichokirin	Type 1	[82]
**Euphorbiaceae**				
*Croton tiglium*	seeds	crotin I, II (2,3)	Type 1	[38]
*Gelonium multiflorum*	seeds	gelonin	Type 1	[74]
*Hura crepitans*	latex	*Hura crepitans* RIP	Type 1	[72]
*Hura crepitans*	latex	*Hura crepitans* lectin	Type 2	[60]
*Jatropha curcas*	seeds	curcin 2	Type 1	[38]
*Manihot palmata*	seeds	mapalmin	Type 1	[75]
*Manihot utulissima*	seeds	manutin 1, 2	Type 1	[83]
**Iridaceae**				
*Iris hollandica*	bulbs	*Iris* RIP A1, A2, A3	Type 1	[84]
*Iris hollandica*	bulbs	IRA	Type 2	[62]
**Lamiaceae**				
*Clerodendrum inerme*	leaves	CIP-29, -34	Type 1	[85]
**Nyctaginaceae**				
*Bougainvillea spectabilis*	leaves	bouganin	Type 1	[73]
*Mirabilis jalapa*	seeds	MAP S	Type 1	[86]
**Passifloraceae**				
*Adenia digitata*	roots	modeccin	Type 2	[50]
*Adenia volkensii*	roots	volkensin	Type 2	[61,87]
*Adenia stenodactyla*	caudices	stenodactylin	Type 2	[64,65]
*Adenia lanceolata*	caudices	lanceolin	Type 2	[64,65]
**Phytolaccaceae**				
*Phytolacca americana*	seeds	PAP-S1, -S2	Type 1	[88]
*Phytolacca americana*	cell culture	PAP-C	Type 1	[89]
*Phytolacca americana*	roots	PAP-R	Type 1	[75]
*Phytolacca dioica*	leaves	PD-L1,-L2, -L3, -L4	Type 1	[90]
*Phytolacca dioica*	seeds	PD-S1, -S2, -S3	Type 1	[91]

**Table 2:** Main immunotoxins purified and characterized by Stirpe and co-workers.

Immunotoxin	Molecular Target	Disease	Ref.
anti-Thy1.1 mAb/gelonin	CD90	Lymphoma	[95]
RSVE/PAP, RSVE/gelonin, RSVE/MCI	Sendai virus receptors	All cells	[93]
anti-Thy1.1 mAb/saporin	CD90	Lymphoma	[96]
anti-Id-1/saporin	Surface IgM idiotype of B-cell lymphoma	Lymphoblastic leukaemia	[109,110]
Campath 1/saporin:	CD52	T-lymphocytes	[111]
anti-saporin/ anti-Id-1 bispecific mAb	Surface IgM idiotype of B-cell lymphoma	Lymphoma	[98]
anti-Thy1.1 mAb/bryodin anti-Thy1.1 mAb/momordin	CD90	Lymphoma	[97]
8A/saporin, 62B1/saporin	Plasma cell antigens	Myeloma	[112]
TEC-T4/saporin	CD4	T-lymphocytes	[113]
8A/momordin	Plasma cell antigens	Myeloma	[114]
8A/saporin, 62B1/saporin, 8A/momordin	B-cell antigens	Myeloma	[115]
8A/momordin	B-cell antigens	Ex vivo bone marrow purging, MM	[106]
MRK16+anti-mouse/saporin	gp 170 surface epitope	Colon carcinoma	[116]
anti-CD2/saporin, UCHT1/saporin, anti-CD5/saporin, anti-CD25/saporin, C11/saporin	CD2, CD3, CD5, CD25, CD45	PHA-stimulated T lymphocytes	[117]
BerH2/saporin, BerH2/dianthin30, BerH2/momordin, BerH2/ricin A chain, BerH2/PAP-S	CD30	HL	[102,104,105,108,118,119,120,121,122,123]
anti-CD4, anti-CD30, C11, 8A, 62B1+anti-mouse IgG/saporin	CD4, CD30, CD45, plasma cell antigens	B- and T-lymphoma, MM	[124]
B-B10/saporin	CD25	GvHD	[125]
ML30/saporin	HSP-65	HSP-65^+^ tumours	[126]
ML30+anti-mouse IgG/saporin	HSP-65	HSP-65^+^ tumours	[127]
anti-CD5/momordin	CD5	T-lymphocytes leukaemia, lymphoma	[128]
anti- saporin/anti-CD25 bispecific mAb	CD25	Lymphocytes, leukaemia, lymphoma	[129]
GT2/saporin, OKT11/saporin, 8E5B3/saporin, 8G5B12/saporin, 7A10C9/saporin	CD2	T-lymphocytes, leukaemia, lymphoma	[130,131]
anti-saporin/anti-CD22, anti-saporin/anti-CD38 bispecific mAbs	CD22CD38	Lymphocytes, leukaemia, lymphoma	[132]
anti-gelonin/anti-CD30 bispecific mAb	CD30	HL	[133]
anti-saporin/antiCD25, anti-saporin/anti-CD30, anti-gelonin/anti-CD30 bispecific mAbs	CD25, CD30	HL	[134]
48–127 mAb/momordin I 48–127 mAb/PAP-S 48–127 mAb/saporin-S6	gp54	Bladder tumours	[135]
B-B2/saporin B-B4/saporin	Myeloma antigens	MM	[136]
Mint5/ocymoidine Mint5/pyramidatine	EGFR	EGFR over-expressing tumours	[137]
OM124/saporin OM124/momordin OM124/PAP-S	CD22	NHL	[138]
antiCD80/saporin, bouganin, gelonin antiCD86/saporin, bouganin, gelonin	CD80CD86	HL, CD80/CD86^+^ tumours	[139]
scFv#83/saporin scFv#40/saporin scFv#67/saporin	CTLA-4	Activated T cells, transplant rejection, GvHD, leukaemia	[140,141,142,143]
mAb73/saporin	AChR	Oculo-motility disorders	[144]
Rituximab/saporin	CD20	NHL	[145]
IB4/saporin	CD38	Haematological CD38^+^ neoplasia	[146]

Abbreviations: EGFR, epidermal growth factor receptor; gp, glycoprotein; GvHD, Graft versus Host Disease; HL, Hodgkin’s Lymphoma; HSP, heat shock protein; MM, multiple myeloma; NHL, Non-Hodgkin’s Lymphoma; PHA, Phytohaemagglutinin.
